# Characterizing the replicability of cell types defined by single cell RNA-sequencing data using MetaNeighbor

**DOI:** 10.1038/s41467-018-03282-0

**Published:** 2018-02-28

**Authors:** Megan Crow, Anirban Paul, Sara Ballouz, Z. Josh Huang, Jesse Gillis

**Affiliations:** Cold Spring Harbor Laboratory, One Bungtown Road, Cold Spring Harbor, NY 11724 USA

## Abstract

Single-cell RNA-sequencing (scRNA-seq) technology provides a new avenue to discover and characterize cell types; however, the experiment-specific technical biases and analytic variability inherent to current pipelines may undermine its replicability. Meta-analysis is further hampered by the use of ad hoc naming conventions. Here we demonstrate our replication framework, MetaNeighbor, that quantifies the degree to which cell types replicate across datasets, and enables rapid identification of clusters with high similarity. We first measure the replicability of neuronal identity, comparing results across eight technically and biologically diverse datasets to define best practices for more complex assessments. We then apply this to novel interneuron subtypes, finding that 24/45 subtypes have evidence of replication, which enables the identification of robust candidate marker genes. Across tasks we find that large sets of variably expressed genes can identify replicable cell types with high accuracy, suggesting a general route forward for large-scale evaluation of scRNA-seq data.

## Abstract (web summary)

Single cell RNA-sequencing analysis poses challenges in replication due to technical biases and analytic variability among bioinformatics pipelines. Here, Crow et al develop MetaNeighbor for measuring cell-type replication across datasets, and use it to identify marker genes for neuron subtypes with evidence of replication.

## Introduction

Single-cell RNA-sequencing (scRNA-seq) has emerged as an important new technology enabling the dissection of heterogeneous biological systems into ever more refined cellular components. One popular application of the technology has been to try to define novel cell subtypes within a tissue or within an already refined cell class, as in the lung^1^, pancreas^2–5^, retina^6,7^, or others^8–10^. Because they aim to discover completely new cell subtypes, the majority of this work relies on unsupervised clustering, with most studies using customized pipelines with many unconstrained parameters, particularly in their inclusion criteria and statistical models^7,8,11,12^. While there has been steady refinement of these techniques as the field has come to appreciate the biases inherent to current scRNA-seq methods, including prominent batch effects^13^, expression drop-outs^14,15^, and the complexities of normalization-given differences in cell size or cell state^16,17^, the question remains: how well do novel transcriptomic cell subtypes replicate across studies?

In order to answer this, we turned to the issue of cell diversity in the brain, a prime target of scRNA-seq as deriving a taxonomy of cell types has been a long-standing goal in neuroscience^18^. Already more than 50 single-cell RNA-seq experiments have been performed using mouse nervous tissue (e.g., ref. ^19^) and remarkable strides have been made to address fundamental questions about the diversity of cells in the nervous system, including efforts to describe the cellular composition of the cortex and hippocampus^11,20^, to exhaustively discover the subtypes of bipolar neurons in the retina^6^, and to characterize similarities between human and mouse midbrain development^21^. This wealth of data has inspired attempts to compare data^6,12,20^ and more generally there has been a growing interest in using batch correction and related approaches to fuse scRNA-seq data across replicate samples or across experiments^6,22,23^. Historically, data fusion has been a necessary step when individual experiments are underpowered or results do not replicate without correction^24–26^, although even sophisticated approaches to merge data come with their own perils^27^. The technical biases of scRNA-seq have motivated interest in correction as a seemingly necessary fix, yet evaluation of whether results replicate remains largely unexamined, and no systematic or formal method has been developed for accomplishing this task.

**Fig. 1:**
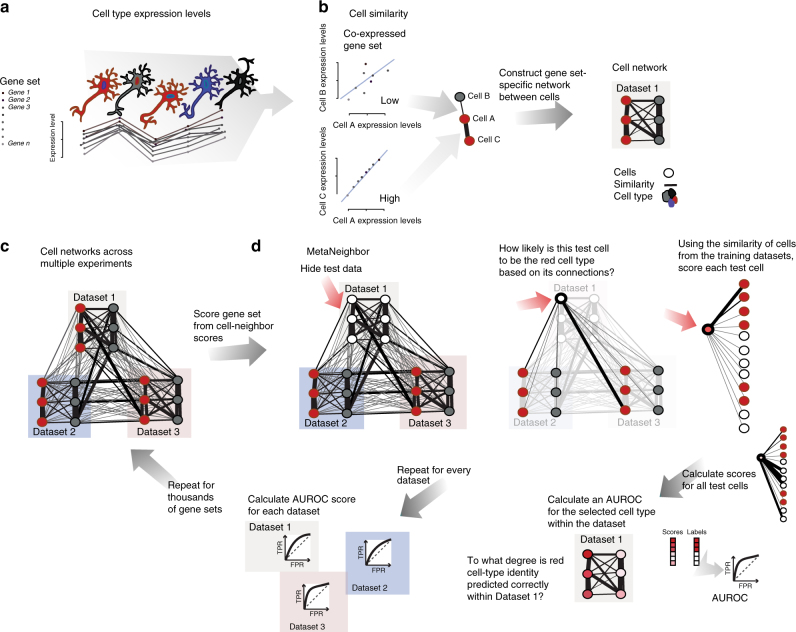
MetaNeighbor quantifies cell-type identity across experiments. **a** Schematic representation of gene set co-expression across individual cells. Cell types are indicated by their color. **b** Similarity between cells is measured by taking the correlation of gene set expression between individual cells. On the top left of the panel, gene set expression between two cells, A and B, is plotted. There is a weak correlation between these cells. On the bottom left of the panel we see the correlation between cells A and C, which are strongly correlated. By taking the correlations between all pairs of cells we can build a cell network (right), where every node is a cell and the edges represent how similar each cell is to each other cell. **c** The cell network that was generated in **b** can be extended to include data from multiple experiments (multiple datasets). The generation of this multi-dataset network is the first step of MetaNeighbor. **d** The cross-validation and scoring scheme of MetaNeighbor is demonstrated in this panel. To assess cell-type identity across experiments we use neighbor voting in cross-validation, systematically hiding the labels from one dataset at a time for testing. Cells within the test set are predicted as similar to the cell types from other training sets using a neighbor-voting formalism. Whether these scores prioritize cells as the correct type within the dataset determines the performance, expressed as the AUROC. In other words, comparative assessment of cells occurs only within a dataset, but is based only on training information from outside that dataset. This is then repeated for all gene sets of interest

To address this gap in the field, we propose a simple, supervised framework, MetaNeighbor (meta-analysis via neighbor voting), to assess how well cell-type-specific transcriptional profiles replicate across datasets. Our basic rationale is that if a cell type has a biological identity rooted in the transcriptome, then knowing its expression features in one dataset will allow us to find cells of the same type in another dataset. We make use of the cell-type labels supplied by data providers, and assess the correspondence of cell types across datasets by taking the following approach (see schematic, Fig. 1):

1. We calculate correlations between all pairs of cells that we aim to compare across datasets based on the expression of a set of genes. This generates a network where each cell is a node and the edges are the strength of the correlations between them.
2. Next, we do cross-dataset validation: we hide all cell-type labels (“identity”) for one dataset at a time. This dataset will be used as our test set. Cells from all other datasets remain labeled, and are used as the training set.
3. Finally, we predict the cell-type labels of the test set: we use a neighbor-voting algorithm to predict the identity of the held-out cells based on their similarity to the training data.

Conceptually, this resembles approaches for the validation of sample clustering^28,29^, which have primarily been applied to compare microarray results with respect to tumor subtyping^30,31^. Our method builds on these ideas, adapting and applying them for the first time to the question of cell identity in scRNA-seq, and specifically exploiting the patterns of co-expression believed to drive results^32^. Because our implementation is extremely fast, this approach readily permits carefully defined control experiments to investigate the data features that drive high performance, such as the dependence on expression variability, gene set size, rarity of cell types, or subtlety of transcriptional identity.

We evaluate the replicability of cell-type transcriptional identity by taking sequential steps according to the basic taxonomy of brain cells: first classifying neurons vs. non-neuronal cells across eight scRNA-seq studies, then classifying cortical inhibitory neurons vs. excitatory neurons, and for our final step, we align interneuron subtypes across three studies. With detailed control experiments and empirical modeling, we validate the use of HVGs for cross-dataset cell identification, a common approach for feature selection within individual experiments^4,33–35^. Testing hundreds of gene sets, we find strong replication of neuronal identity when compared to non-neurons, and excitatory vs. inhibitory neurons, even across widely varying techniques such as nuclear RNA-sequencing or Drop-seq. Furthermore, we readily identify 11 interneuron subtypes that appear to replicate across datasets, including Chandelier cells and five novel subtypes defined by transcriptional clustering in previous work. Meta-analysis of differential expression across these highly replicable interneuron subtypes correctly identified canonical marker genes, as well as new candidates that may be used for improved molecular genetic targeting and to understand the diverse phenotypes of these cells.

## Results

### Assessing neuronal identity with MetaNeighbor

We aimed to measure the replicability of cell identity across tasks of varying specificity. Broadly, these are divided into tasks where we are recapitulating known cell identities, and ones where we are measuring the replicability of novel cell identities discovered in recent research. The former is the focus of this subsection: first, by assessing how well we could distinguish neurons from non-neuronal cells (“task one”), and next assessing the discriminability of excitatory and inhibitory neurons (“task two”). As detailed in the Methods, MetaNeighbor outputs a performance score for each gene set and task. This score is the mean area under the receiver operator characteristic curve (AUROC) across all folds of cross-dataset validation, and it can be interpreted as the probability that we will rank a cell of the type we aim to characterize (a “positive”) higher than a cell from the out-group (a “negative”). For example, given information from a training dataset that labels neurons as positives and non-neurons as negatives and asking the algorithm to identify neurons within a testing dataset, the AUROC is the probability a neuron will be ranked above a non-neuron. The AUROC varies between 0 and 1, with 1 being perfect classification, 0.5 meaning that we have performed as well as if we had randomly guessed the cell’s identity (null), and 0.9 or above being extremely high. Low scores (0–0.3) can be interpreted with as much confidence as high scores, and mean that, for example, a neuron is definitely not a non-neuron. Comparison of scores across gene sets allows us to discover their relative capacity to discriminate cell types.

**Fig. 2:**
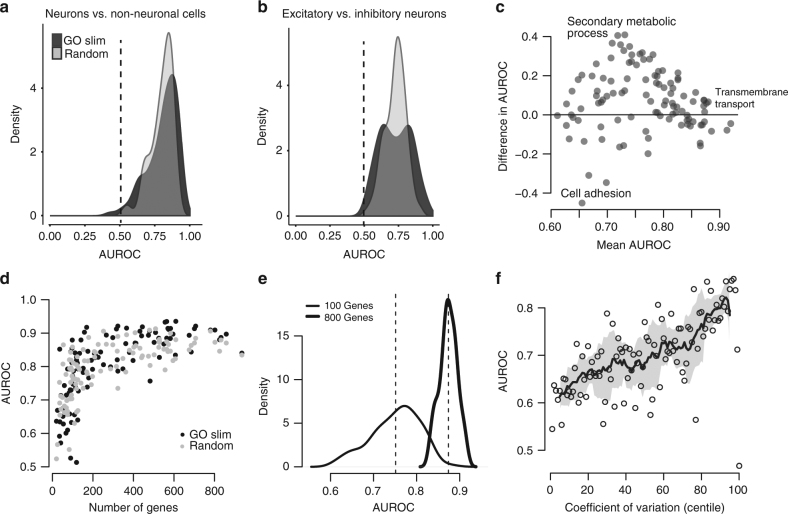
Cell-type identity is widely represented in the transcriptome. **a**,**b** Distribution of AUROC scores from MetaNeighbor for discriminating neurons from non-neuronal cells (“task one”, **a**) and for distinguishing excitatory vs. inhibitory neurons (“task two”, **b**). GO scores are in black and random gene set scores are plotted in gray. Dashed gray lines indicate the null expectation for correctly guessing cell identity (AUROC = 0.5). For both tasks, almost any gene set can be used to improve performance above the null, suggesting widespread encoding of cell identity across the transcriptome. **c** Comparison of GO group scores across tasks. GO groups at the extremes of the distribution are labeled. Most gene sets have higher performance for task one, and a number of groups have high performance for both tasks (e.g., transmembrane transport). **d** Task one AUROC scores for each gene set are plotted with respect to the number of genes. A strong, positive relationship is observed between gene set size and AUROC score, regardless of whether genes were chosen randomly or based on shared functions. **e** Distribution of AUROC scores for task one using 100 sets of 100 randomly chosen genes, or 800 randomly chosen genes. The mean AUROC score is significantly improved with the use of larger gene sets (mean 100 = 0.80 ± 0.05, mean 800 = 0.90 ± 0.03). **f** Relationship between AUROC score and coefficient of variation. Points indicate individual gene set performances, the line shows the running average and the SD of the line is indicated by the shaded region. Task one was re-run using sets of genes chosen based on mean coefficient of variation across datasets. A strong positive relationship was observed between this factor and performance (*r*_s_ ~ 0.67)

As described above, in task one we assessed how well we could identify neurons and non-neuronal cells across eight datasets^7,11,20,36–40^ with a total of 13,928 cells (Supplementary Table 1). Although this was designed to be fairly simple, we were interested to discover that AUROC scores are significantly higher than chance for all gene sets tested, including all randomly chosen sets (mean AUROC ± SD reported throughout; AUROC = 0.80 ± 0.1, Fig. 2a). A bootstrapped sampling of the datasets showed a trend toward increased performance with the inclusion of additional training data, indicating that we are recognizing an aggregate signal across datasets (Supplementary Fig. 1). However, the significant improvement of random sets over the null (i.e., AUROC = 0.5) means that prior knowledge about gene function is not required to differentiate between these cell classes. Randomly chosen sets of genes have decidedly non-random expression patterns that enable discrimination between these cell types. This is particularly surprising in the context of cross-dataset assessment, where the low dimensionality of cell identity observed within laboratories^41^ is confounded by the even lower dimensionality of experimental identity. This result recalls the startling finding by Venet et al. that “most random gene expression signatures are related to breast cancer outcome”^42^; cell identity appears to be as clearly ascertainable.

Task two aimed to assess how well we could discriminate between cortical excitatory and inhibitory neurons across four studies with a total of 2809 excitatory and 1162 inhibitory neurons^11,12,20,36^. Similar to our previous results, we saw that AUROC scores are significantly higher than chance (AUROC = 0.69 ± 0.1, Fig. 2b). While performance is high for both tasks, it is unclear whether the same gene sets are useful for distinguishing between neurons and non-neurons and between excitatory and inhibitory neurons. Comparing GO group performance we find that a handful of gene sets have high performance for both tasks (e.g., GO:0055085 transmembrane transport, AUROC > 0.85, Fig. 2c), while many GO groups show divergent performance. For example, we find that GO:0019748 (secondary metabolic process) is only useful for distinguishing between neurons and non-neurons, but not at all for distinguishing between the two neuron classes, perhaps because of cell cycling among non-neuronal cells. On the other extreme, we find that GO:0040011 (cell adhesion) is only useful for distinguishing between neuron classes but not between neurons and non-neuronal cells, which is in line with previous work that found that cell adhesion factors show neuron-type-specific expression^43,44^. These results indicate some degree of functional specificity for gene set performance, but the near equivalent performance of randomly chosen gene sets suggests that transcriptional differences are likely to be encoded in a large number of genes, in line with previous observations^45^. The properties of high-performing sets are investigated in the following section.

### Characterizing features associated with high performance

Consistent with the view that a large fraction of transcripts are useful for determining cell identity, we find a positive dependency of AUROC scores on gene set size, regardless of whether genes within the sets are randomly selected or share some biological functions (Fig. 2d). This was further supported by a comparison of scores for task one when using randomly chosen sets of genes constrained to a given size. Here we used set sizes of 100 or 800, similar to the extremes of the distribution of set sizes used in the GO analysis. AUROC score distributions and means are significantly different between gene sets of different sizes: sets of 100 genes have lower scores but higher variability in performance, whereas sets of 800 genes are more restricted in variance and have higher performance on average (Fig. 2e, AUROC_100_ = 0.75 ± 0.06, AUROC_800_ = 0.87 ± 0.02, *P* < 2.2E−16, Wilcoxon rank-sum test). The variability in performance observed while keeping set size constant suggests that even in random sets there are transcriptional features that contribute to cell identity. We delved into this further by comparing AUROC scores across gene sets chosen based on coefficient of variation, as MetaNeighbor relies on co-variation between genes to detect differences in cell-type profiles. Performing task one again, we find a strong positive relationship between variance and our ability to classify cells (Fig. 2f, Spearman correlation coefficient = 0.67), although interestingly, genes in the top centile are completely uninformative (AUROC = 0.47). Taken together, these observations support the idea that transcriptional identity is broadly encoded across many genes, and suggests that it should be straightforward to select an informative gene set that takes advantage of properties associated with high performance. Testing our capacity to detect and exploit this signal requires us to refine the cell classes that we are characterizing, ideally beyond what is present in existing data to anticipate a wide range of use cases.

### Empirical modeling to determine precision

**Fig. 3:**
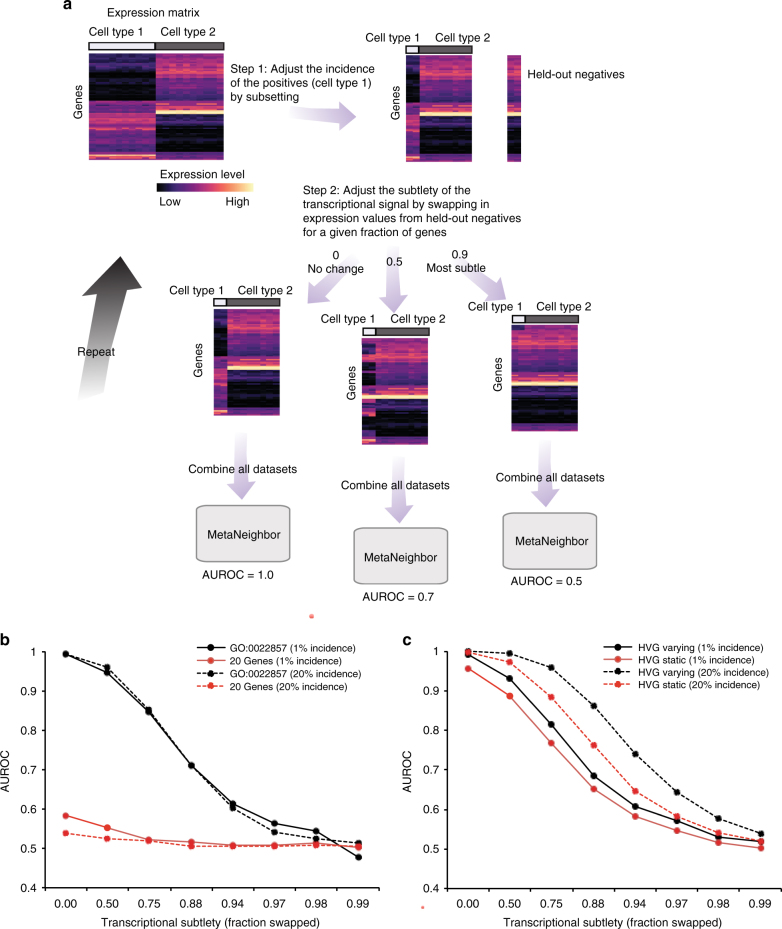
Empirical modeling demonstrates that MetaNeighbor readily identifies rare and transcriptionally subtle cell types. **a** Schematic of the empirical model. For simplicity only a single dataset is depicted. (Top left) In this dataset, we begin with an expression matrix containing gene expression levels for two cell types comprising 10 cells each. Here we will be assessing the replicability of cell type 1 (“positives”) relative to cell type 2 (“negatives”). (Top right) We first adjust cell rarity by randomly sampling subsets of the original expression matrix. In the schematic, incidence is set to 20% (two positives and eight negatives). In addition, we partition two negatives from the original data for later use. (Middle) Next, we adjust transcriptional subtlety by randomly sampling genes from a given fraction of the transcriptome. Gene expression in the positives will be replaced with data from the unused negatives, creating a modeled cell type varying from the negative class only in a subset of its genes. (Bottom) All datasets are combined and MetaNeighbor is run to assess the replicability of the positives at each level of rarity and subtlety. **b** MetaNeighbor results for empirical modeling of excitatory neuron rarity and subtlety, repeated 100 times. Mean performance for the top GO group is in black; performance for 20 randomly chosen genes is shown in red; dashed lines indicate 20% rarity and solid lines show 1% rarity. MetaNeighbor is robust to differences in cell rarity, and can reliably distinguish between types even when they are very similar (AUROC > 0.7 at >88% subtlety). **c** MetaNeighbor results for empirical modeling of excitatory neuron rarity and subtlety using highly variable genes (HVGs), repeated 100 times. Performance for the HVG varying set is shown in black, performance for the HVG static is shown in red; dashed lines indicate 20% rarity and solid lines show 1% rarity. HVGs allow for robust identification of positives even when cells are rare or differences are subtle

Our ultimate aim is to identify all replicable cell types across datasets, some of which may be rare and/or only subtly different from other cell types. To assess the ability of MetaNeighbor to identify cell types in these more realistic scenarios, we set up an empirical model for cell-type rarity and subtlety (Schematic Fig. 3a), using inhibitory and excitatory neuron datasets with >100 cells for each type as these allow us to model cell-type incidence down to 1%^11,12,20^. We define a rare cell type as one in which only a few differing cells are present, and a subtle transcriptional identity is one in which only a few differing genes are present. Thus, to address the impact of rarity on MetaNeighbor’s performance, we alter the incidence of excitatory neurons to be within our observed range of subtype incidences, repeatedly sampling different combinations of cells to obtain mean performance estimates. Subtlety is modeled by swapping out gene information, e.g., the same 95% of the transcriptional profiles across all excitatory cell transcriptional data for data from inhibitory cells, so that all cells sample from the same cell class for 95% of their profile (sampled without replacement to ensure there are no confounding overlaps). At each level of rarity and subtlety we measure AUROCs across datasets with MetaNeighbor, using the highest-performing GO group for these data as a positive control for gene set selection (GO:0022857) and a randomly chosen set of 20 genes as a negative control, having established that small gene sets tend to have low performance.

As expected, GO:0022857 performance is higher than the random set of 20 genes at both 1 and 20% incidences (Fig. 3b). Importantly, MetaNeighbor performance is nearly unaffected by differences in rarity: GO set performance is equally high when excitatory neurons make up 1 or 20% of all cells in each dataset, with *n* as low as 1 cell in the tested data. Of interest is the robustness of MetaNeighbor to transcriptional subtlety. Of course, increasing subtlety leads to worse performance at both incidences, and falls to chance levels at subtleties >99% (AUROC = 0.5). However, even at almost 90% subtlety MetaNeighbor correctly identifies excitatory neurons with a mean AUROC of 0.71. Since this subtlety is relative to the transcriptional variability that exists between inhibitory and excitatory cells, it is quite extreme. Consistent with our previous results comparing performance across all GO functions, this suggests that there are marked and widespread differences in excitatory and inhibitory neuron gene expression, such that even sampling a small fraction of genes (<10%) allows for identification of these two classes. In sum, these results provide strong evidence that MetaNeighbor is robust to differences in rarity, and gives guidance for the interpretation of AUROC scores in light of this factor, suggesting the subtlety of cell identity relative to the outside control.

### Empirical modeling to evaluate gene set selection

In the previous section we demonstrated that the highest-performing GO group is robust to variation in either incidence or transcriptional subtlety, still permitting cell identification when cells are rare or only subtly distinguishable. Determining this gene set requires known concordance of cell types across datasets. When concordance is unknown, for example, when cell-type labeling is idiosyncratic, it is necessary to have a strategy to identify informative gene sets ab initio. Expert knowledge of marker genes is one possibility, although this approach may not be extensible to newly described cell subtypes and suffers from potential ascertainment bias. As a more general alternative, the selection of highly variable genes (HVGs) is commonly used in a single-cell analysis prior to dimension reduction and clustering^4,7,33–35^, as it is thought that differentially expressed genes or marker genes should be preferentially variable, and less subject to joint low-level noise. This is in line with our previous observation that gene sets containing HVGs are high-performing. Indeed, when we select a set of HVG (detailed in Methods) we can almost perfectly identify excitatory neurons compared to inhibitory neurons across datasets (AUROC = 0.99), which is equivalent to the highest-performing GO group, but without any prior knowledge.

In parallel to our previous analyses, we assessed the robustness of HVG selection at different levels of rarity and subtlety, using either HVG picked from the original dataset that includes all cells (HVG static), or HVG re-calculated based on the precise subset of data included in each run of the empirical model (HVG varying; Fig. 3c). Here, we see that our HVG selection strategy performs equally to or better than the highest-performing GO group for both rare cell types (1–20% of total), and for subtle cell types (differing from out-group by <10%). Interestingly, the HVG heuristic is even responsive to the precise data sampling, yielding modestly improved performance when it is selected based on the precise data generated by the empirical model.

These results provide evidence that MetaNeighbor can readily identify cells of the same type across datasets, without relying on specific knowledge of marker genes, even when cells are rare or only subtly different from the out-group. Importantly, these results also provide guidelines for interpreting AUROCs at cell incidences ≥1% in terms of their implications for the promiscuity of cell identity across the transcriptome.

### Investigating interneuron subtypes with MetaNeighbor

Cortical inhibitory interneurons have diverse characteristics based on their morphology, connectivity, electrophysiology, and developmental origins, and it has been an ongoing goal to define cell subtypes based on these properties^18^. In a related paper^44^, we describe the transcriptional profiles of GABAergic interneuron types, which were targeted using intersectional marker gene expression, cell lineage, laminar distribution, and birth timing, and have been extensively phenotyped both electrophysiologically and morphologically^46^. Previously, two studies were published in which new interneuron subtypes were defined based on scRNA-seq transcriptional profiles^11,20^. Because of differences in experimental design and analytic choices, the two studies found different numbers of subtypes (16 in one and 23 in the other). The authors of the later paper compared their outcomes by looking at the expression of a handful of marker genes, which yielded mixed results: a small number of cell types seemed to have a direct match but for others the results were more conflicting, with multiple types matching to one another, and others having no match at all. Here we aimed to more quantitatively assess the similarity of their results, and compare them with our own data that derives from phenotypically characterized subpopulations; i.e., not from unsupervised expression clustering (see Supplementary Table 2 for sample information).

**Fig. 4:**
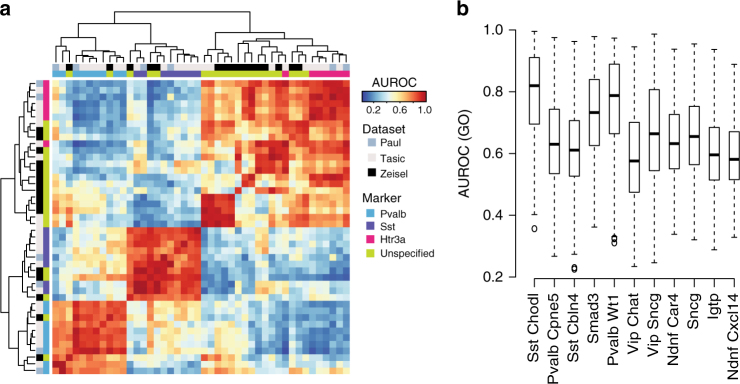
Cross-dataset analysis of interneuron diversity. **a** Heatmap of AUROC scores between interneuron subtypes based on the highly variable gene set (HVG). Dendrograms were generated by hierarchical clustering of Euclidean distances using average linkage. Row and column colors indicate data origin and marker expression. Clustering of AUROC score profiles recapitulates known cell-type structure, with major branches representing the Pv, Sst, and Htr3a lineages. **b** Boxplots of GO performance (3888 sets) for each putatively replicated subtype, ordered by their AUROC score from the highly variable gene set. Subtypes are labeled with the names from Tasic et al. A positive relationship is observed between AUROC scores from the highly variable set and the average AUROC score for each subtype

To examine how the previously identified interneuron subtypes are represented across the three studies, we tested the similarity of each pair of subtypes across datasets using HVGs. This was done by alternately considering each subtype as the positive training set, and each other subtype as the positive test set, answering questions of the class, e.g., “How well does the Zeisel_Int1 HVG expression predict the identity of the Tasic_Smad3 subtype relative to all interneurons in the Tasic data?”. Each subtype ranges in incidence from 1 to 24% of the total number of cells within its own dataset, well within the range of the sensitivity of MetaNeighbor as established above. Corroborating earlier results, we find that for each interneuron type profiled by Paul et al. we find a reciprocal best match in the pre-existing data (Table 1, all scores in Supplementary Data 1). In addition, expanding our criteria to include all reciprocal best matches, and those with AUROC scores ≥0.95, we find additional matches for the Paul subtypes, as well as correspondence among five subtypes that were assessed only in the Tasic and Zeisel data. Overall, we identified 11 subtypes representing 24/45 (53%) types (Fig. 4a), with total *n* for each subtype ranging from 25 to 189 out of 1583 interneurons across all datasets (1.5–11%). Our corresponding subtypes also confirm the marker gene analysis performed by Tasic et al. (Supplementary Data 1), without requiring manual gene curation. Because we quantify the similarity among types, we can prioritize matches and use these as input to MetaNeighbor for further evaluation.

**Table 1:** Putatively replicated interneuron subtypes and their MetaNeighbor scores for HVG and top GO groups

Interneuron subtype (author, label)	AUROC HVG	AUROC Top GO	Top GO group	Description
Tasic, Sst Chodl Paul, Som-Nos1 Zeisel, Int1	1.00	1.00	GO:0071407	Cellular response to organic cyclic compound
Tasic, Pvalb Cpne5 Paul, ChC	0.99	0.98	GO:0043005	Neuron projection
Tasic, Sst Cbln Paul, Sst-CR	0.98	0.96	GO:0090257	Regulation of muscle system process
Tasic, Smad3 Zeisel, Int14	0.97	0.98	GO:0007169	Transmembrane receptor protein tyrosine kinase signaling pathway
Tasic, Pvalb Wt1 Paul, Pv	0.96	0.97	GO:0045202	Synapse
Tasic, Vip Chat Paul, Vip-CR Zeisel, Int10	0.96	0.95	GO:0007188	Adenylate cyclase-modulating G-protein-coupled receptor signaling pathway
Tasic, Vip Sncg Paul, Vip-Cck	0.95	0.99	GO:0097458	Neuron part
Tasic, Sncg Zeisel, Int6	0.95	0.95	GO:0097458	Neuron part
Tasic, Ndnf Car4 Zeisel, Int15	0.95	0.94	GO:0007610	Behavior
Tasic, Igtp Zeisel, Int13	0.94	0.94	GO:0010975	Regulation of neuron projection development
Tasic, Ndnf Cxcl14 Zeisel, Int12	0.91	0.89	GO:0007611	Learning or memory

Subtypes are listed from highest to lowest AUROC using the HVGs. HVGs perform comparably to or better than the top-ranking GO group for most subtypes

To assess cell identification more broadly, we ran MetaNeighbor with these new across-dataset subtype labels, measuring predictive validity across all gene sets in GO (Fig. 4b). The distribution of AUROC scores varies across subtypes but we find that the score from the high variability gene set is representative of overall trends, with high-performing groups showing higher mean AUROC scores over many gene sets. Both the high mean AUROCs across all putative replicate subtypes, and the similarity of maximum performance suggest that distinctive gene co-expression can be observed in each subtype (maximum AUROC = 0.92 ± 0.04). As with previous tasks, we find little difference between functional gene sets and random sets (AUROC_Random_ = 0.67 ± 0.06, AUROC_GO_ = 0.68 ± 0.1). Top performing GO groups for each of the 11 replicate interneuron subtypes are primarily related to neuronal function, which is expected because of the large size of these gene sets and their likelihood of expression and variation in these cells (Table 1).

These results suggest that HVG sets can be used alongside pairwise testing and training as a heuristic to identify replicable subtypes for further evaluation. Indeed, while outside the scope of our primary analysis, we have found that re-analysis of tens of thousands of cells from mouse cortical and hippocampal pyramidal neurons^11,12,20^, retina^6,7^, and human pancreas^2,3,5,47,48^ provide strong evidence for the broad applicability of this approach (Supplementary Fig. 2, Supplementary Data 2, and Supplementary Note 1).

### Identifying subtype-specific genes

**Fig. 5:**
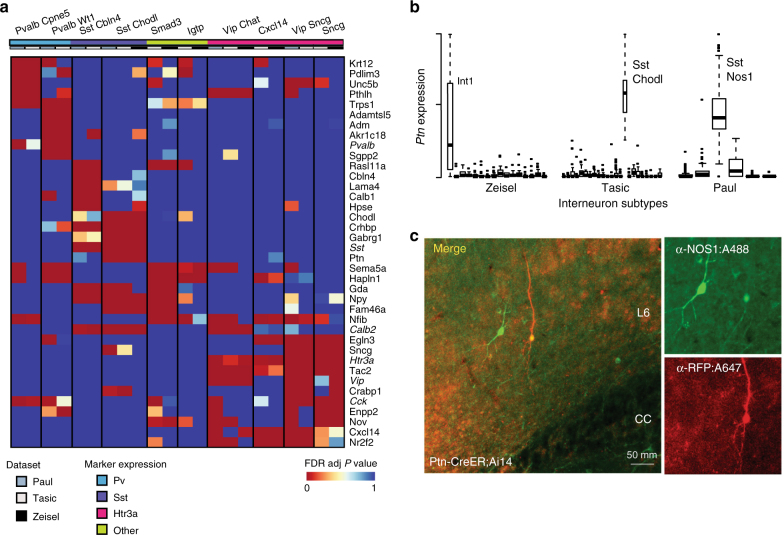
Replicated subtypes show consistent differential expression. **a** (Top) Heatmap of FDR-adjusted *P *values of top differentially expressed genes among replicated interneuron subtypes (NB only 10 subtypes are shown as no differentially expressed genes were found for the Ndnf Car4 subtype). Subtype names are listed at the top of the columns and are labeled as in Tasic et al^20^. Many genes are commonly differentially expressed among multiple subtypes, but combinatorial patterns distinguish them. **b** Standardized *Ptn* expression is plotted across the three experiments, where each box represents an interneuron subtype. Boxes bound the quartiles, middle lines represent the median, whiskers extend to 1.5 times the interquartile range, and values outside of this range are shown as individual points. High, but variable expression is observed across the three Sst Chodl types. **c** Confocal images of co-immunostaining for *Ptn*-CreER;Ai14 with RFP and Nos1 antibodies in adult mouse cortex. *Ptn*-CreER;Ai14 expression was induced with low-dose tamoxifen postnatally. Clear co-labeling is observed in a deep layer (L6) long projecting neuron

ScRNA-seq experiments often seek to define marker genes for novel subtypes, although typically only a small number of genes are reported in single-cell papers because of the complexity of discussing dozens of cell types as well as the potential technical confounds, which would limit the expected replicability of a more comprehensive list^5,7,11,20^. Here we aimed to identify possible marker genes that would allow discrimination among interneuron subtypes. For each of our identified replicate subtypes we generated a ranked list of possible marker genes by performing differential expression analysis within each study (e.g., Int1 vs. all other interneurons in the Zeisel study) and combining *P* values across studies using Fisher’s method (Supplementary Data 3). Figure 5a shows the FDR-adjusted *P* values for the top candidates based on fold change for the 10 replicated interneuron subtypes with overlapping differential expression patterns. The majority of these genes have previously been characterized as having some degree of subtype-specific expression, for example, we readily identify genes that were used for the Cre-driver lines in the Tasic and Paul studies (*Sst, Pvalb, Vip, Cck*, and *Htr3a*), as well as all markers previously reported to intersect between the Tasic and Zeisel data (Supplementary Data 3). Even though we filtered for genes with high fold changes, we see that many genes are differentially expressed in more than one subtype. Notably, considerable overlap can be observed among the *Htr3a*-expressing types. For example, the Vip Sncg subtype is only subtly different from the Sncg subtype across this subset of genes, with the Sncg cells lacking differential expression of *Cxcl14* and *Nr2f2*.

We also identify some novel candidates, including *Ptn*, or pleiotrophin, which is significantly more expressed in the three *Sst* and *Nos1*-expressing subtypes than in the others (Fig. 4b). It is thus expected to be discriminative of these neurons compared to other interneuron types. We validated *Ptn* expression with genetic targeting^44^, and we show clear expression in neurons that stain positively for Nos1 and have morphological features characteristic of long projecting interneurons (Fig. 4c). *Ptn* is a growth factor, and we suggest that its expression may be required for maintaining the long-range axonal connections that characterize these cells. These cells are well described by current markers; however, this approach is likely to be of particular value for novel subtypes that lack markers, allowing researchers to prioritize genes for follow-up by assessing robustness across multiple data sources.

## Discussion

Single-cell transcriptomics promises to have a revolutionary impact by enabling comprehensive sampling of cellular heterogeneity; nowhere is this variability more profound than within the brain, making it a particular focus of both single-cell transcriptomics and our own analysis into its replicability. The substantial history of transcriptomic analysis and meta-analysis gives us guidance about bottlenecks that will be critical to consider in order to characterize cellular heterogeneity. The most prominent of these is laboratory-specific bias, likely deriving from the adherence to a strict set of internal standards that may filter for some classes of biological signal (e.g., poly-A selection) or induce purely technical grouping (e.g., by sequencing depth). Because of this, it is imperative to be able to compare data across studies and determine some form of consensus. Indeed, while this work was under review, five manuscripts became available that tackle different aspects of this problem, including robust low-dimensional representation and the use of reference data for cell classification^49,50^, batch correction using nearest neighbors^22^, and data fusion via manifold alignment^23,51^. These papers collectively add to existing techniques for correcting expression data or predicting sample identity, topics closely related to our own interest. However, all take as their premise that replicable signatures occur and data can either be aligned to obtain them or novel identities predicted to best fit them. Our study is unique in its aim and ability to not only identify replicable cell types but to rigorously quantify the degree to which replicability occurs, making use of interpretable methods and concrete performance metrics. Historically, cross-laboratory validation has been regarded as extremely challenging^52^, but using carefully defined controls, our work demonstrates that single-cell identities exhibit cross-lab replication. Importantly, we do not rely on novel statistical metrics; our work provides a formal means to quantitatively assess cell-type replicability using existing statistical concepts. The essential premise of our method is that if a cell type has a distinct transcriptional profile within a dataset, then an algorithm trained from that dataset will correctly identify the same type within an independent dataset.

The ease-of-use and quantitative output of our approach allowed us to canvas existing data broadly and draw a number of important conclusions. We validated the identity of 11 interneuron subtypes, and described replicate transcriptional profiles to prioritize possible marker genes, including *Ptn*, a growth factor that is preferentially expressed in Sst Chodl cells. One major surprise of our analysis is the degree of replicability in the current data. AUROC scores are exceptionally high, particularly when considered in the context of the well-described technical confounds of single-cell data. We suspect that this reflects the fundamental nature of the biological problem we are facing: cell types can be identified by their transcriptional profiles, and the biological clarity of the problem overcomes technical variation. Echoing earlier work on cancer subtyping^30^, we caution that orthogonal data will be required to more firmly establish the biological basis of cell identity; the current estimates must be regarded as optimistic since most clusters are defined from gene expression to begin with. However, the clarity of cell identity is further suggested by our result that cell identity has promiscuous effects within transcriptional data. While in-depth investigation of the most salient gene functions is required to characterize cell types, to simply identify cell types is relatively straightforward. This is necessarily a major factor in the apparent successes of unsupervised methods in determining novel cell types and suggests that cell-type identity is clearly defined by transcriptional profiles, regardless of cell selection protocols, library preparation techniques, or fine-tuning of clustering algorithms.

Our empirical modeling suggests that this clear signal will permit cell types to be identified down to even greater specificity, but not indefinitely, and some areas of concern within even the present data are worth highlighting. In this work we opted to use the cluster labels provided by the original authors, in essence to characterize both the underlying data as well as current analytic practices. However, this has limitations where studies cluster to different levels of specificity. This reflects quite real ambiguity about the degree of specificity associated with the term “cell type”. For example, nearly all Pvalb subtypes from the Tasic dataset and the Zeisel Int3 type have AUROC scores >0.9 for the Paul Pv type, as can be seen in the bottom left corner of the heatmap in Fig. 4a, suggesting that these may form one larger or more general Parvalbumin-positive type. It is here that the concrete meaning of AUROCs helps. While reciprocal top hits and AUROCs >0.95 reflect extreme confidence in a highly concordant cell type, more moderate scores are still meaningful. In most domains of biological study, AUROCs >0.9 are extraordinarily high (e.g., refs. ^53,54^), and we suggest that any such pairing is worthy of discussion. Moving past this point and distinguishing between only subtly different types will be difficult for any analysis, and their discovery will require consideration of appropriate controls and comparisons. The notion of experimental control is built into our scoring method (AUROCs), which by definition compares positive and negative cases across the data. As in all classification tasks, choice of an unreasonable out-group or control will generate misleading results, and the closest out-group is usually the most appropriate. Within our current framework we suggest that a hierarchical approach, moving from broad to subtle categories, will provide a comprehensive, multiscale view of cell-type replicability. We note that our implementation is both robust and fast, but further development of MetaNeighbor and its basic framework may yield improvements (e.g., optimization of feature selection, multikernel approaches for cell similarity network estimation, more sophisticated machine-learning algorithms).

A key bottleneck, however, is the availability of the data itself. While many groups make their data available in some format, without field-wide standards these data are necessarily more difficult to wrangle than it need be. A common issue is the absence of inferred cell-type labels. While it will likely take time and concerted effort for naming conventions to be established, it is crucial that authors make cell labels publicly available in easy-to-access flat text files along with the final parsed expression data matrix to which those labels were applied (or derived). Our wish list for study metadata would also include standardized reporting of cell viability estimates, cell capture method, library preparation method and batch identifiers, alongside biological covariates such as age, sex, and strain. More comprehensive reporting would allow for deeper evaluation of technical and biological factors that influence single-cell expression results. As the project of assembling a human cell atlas gets underway^55^, we hope that participants continue to learn lessons from MAQC and other large consortia projects, making results quickly and readily available to the public, and recognizing the value of heterogeneity in experimental and computational approaches to generate biologically robust results with independent and replicable evidence.

## Methods

### Public expression data

Data analysis was performed in R using custom scripts^56^. Processed expression data tables were downloaded from GEO directly, and then subset to genes appearing on both Affymetrix GeneChip Mouse Gene 2.0 ST array (902119) and the UCSC known gene list to generate a merged matrix containing all samples from each experiment. The mean value was taken for all genes with more than one expression value assigned. Where no gene name match could be found, a value of 0 was input. We considered only samples that were explicitly labeled as single cells, and removed cells that expressed fewer than 1000 genes with expression >0. Cell-type labels were manually curated using sample labels and metadata from GEO (see Supplementary Table 1 and Supplementary Table 2).

### Gene sets

Gene annotations were obtained from the GO Consortium ‘goslim_generic’ (August 2015). These were filtered for terms appearing in the GO Consortium mouse annotations “gene_association.mgi.gz” (December 2014) and for gene sets with between 20 and 1000 genes, leaving 106 GO groups with 9221 associated genes. Random gene sets were generated by randomly choosing genes with the same set size distribution as GO slim. Gene sets based on coefficient of variation were generated by measuring the coefficient of variation for each gene within each dataset, ranking these lists, and then taking the average across datasets. The average was then binned into centiles. Sets of HVGs were generated by binning data from each dataset into deciles based on expression level, and then making lists of the top 25% of the most variable genes for each decile, excluding the most highly expressed bin. The HVG set was then defined as the intersect of the HVG lists across the relevant datasets. Although this did not occur within our analysis, the use of the intersect is likely to be too stringent as the number of datasets for comparison increases. In this case, a majority rule on the highly variable set across datasets appears to be a practicable strategy. Further commentary regarding highly variable gene set selection may be found in Supplementary Note 2.

### MetaNeighbor

The input to MetaNeighbor is a set of genes, a data matrix, and two sets of labels: one set for labeling each experiment and one set for labeling the cell types of interest. For each gene set, the method generates a cell–cell similarity network by measuring the Spearman correlation between all cells across the genes within the set, and then ranking and standardizing the network so that all values lie between 0 and 1. The use of rank correlations means that the method is robust to any rank-preserving normalization (i.e., log2, TPM, and RPKM). Ranking and standardizing the networks ensures that distributions remain uniform across gene sets, and diminishes the role outlier similarities can play since values are constrained. In previous work we have demonstrated that networks constructed in this way are both robust and highly effective for capturing gene co-expression as evaluated by a variety of machine-learning methods^57^.

The node degree of each cell is defined as the sum of the weights of all edges connected to it (i.e., the sum of the standardized correlation coefficients between each cell and all others), and this is used as the null predictor in the neighbor-voting algorithm to standardize for a cell’s hubness: cells that are generically linked to many cells are preferentially downweighted, whereas those with fewer connections are less penalized. For each cell-type assessment, the neighbor-voting predictor produces a weighted matrix of predicted labels by performing matrix multiplication between the network and the binary vector (0,1) indicating cell-type membership, and then dividing each element by the null predictor (i.e., node degree). In other words, each cell is given a score equal to the fraction of its neighbors, including itself, which are part of a given cell type^58^. Unlike K-Nearest Neighbors, all cells are neighbors to one another to varying degrees (defined by the weighted cell–cell similarity network). For cross-validation, we permute through all possible combinations of leave-one-dataset-out cross-validation, sequentially hiding each experiment’s cell labels in turn, and then reporting how well we can recover cells of the same type as the mean AUROC across all folds. A key difference from conventional cross-validation is that there are no labeled data within the dataset for which predictions are being made. Labeled data come only from external datasets, ensuring that predictions are driven by signals that are replicable across data sources. To improve speed, AUROCs are calculated analytically, where the AUROC for each cell type *j*, is calculated based on the sum of the ranks of the scores for each cell *i* (Ranks_*i*_), belonging to that cell type, ranked out of all cells within the dataset. This can be expressed as follows:

$${\rm AUROC}_j = \mathop {\sum }\limits_i^{\mathrm{N}} \frac{{{\rm Ranks}_i}}{{N \ast N_{{\rm Neg}}}} - \frac{{N + 1}}{{2 \ast N_{\rm Neg}}}$$

where *N* is the number of true-positives (cells of type *j*), and *N*_Neg_ is the number of true-negatives (cells not of type *j*). Thus, the AUROC calculates the probability that the classifier correctly predicts that a cell of type *j* outranks a cell not of type *j* within the test dataset based on similarity to the labeled data in the training dataset(s). Note that for experiments with only one cell type this cannot be computed as there are no true-negatives. For each gene set, AUROCs are reported as averages across all folds of cross-validation (excluding NAs from experiments with no negatives), and the distribution of mean AUROCs across gene sets is plotted. When reporting performance across many gene sets, results are presented as the mean ± SD across gene sets (e.g., AUROC_GO_ = 0.6 ± 0.1). Estimated run times for MetaNeighbor may be found in Supplementary Table 3. 

### Empirical model of cell-type rarity and subtlety

To test the impact of cell-type rarity and transcriptional subtlely on MetaNeighbor performance, we repeated the excitatory vs. inhibitory cell discrimination task using the Tasic, Zeisel, and Habib datasets, which contained >100 cells per cell type, allowing us to assess cell incidences as low as 1%. The essence of the model is to construct a gene-by-cell matrix in which the biclustering problem to identify cell types from their variation in expression would be increasingly challenging, with both a smaller and smaller fraction of cells (rarity) within the minority class and a smaller and smaller fraction of transcripts distinguishing those cells (subtlety). We model this variability in transcriptional subtlety by sampling different fractions of the transcriptome from the minority class; therefore, for example, a dataset could be generated in which only 1% of cells have only 10% of their gene expression values sampled from the minority class with the remainder sampled from the majority class. Each minority class cell’s expression vector would thus be the discrete combination of two real cells, one excitatory and one inhibitory. In all cases, real expression values are used with strict partitioning, e.g., sampling without replacement from expression vectors defining cells. Each analysis for a given value of rarity and subtlety was repeated 100 times and means across random subsamplings of genes and cells are plotted in Fig. 3.

### Identifying putative replicates

In cases where cell identity was undefined across datasets (i.e., cortical interneuron subtypes) we treated each subtype label as a positive for each other subtype, and assessed similarity using HVGs. For example, Int1 from the Zeisel dataset was used as the positive (training) set, and all other subtypes were considered the test set in turn. Mean AUROCs from both testing and training folds are plotted in the heatmap in Fig. 4. Reciprocal best matches across datasets and AUROCs ≥0.95 were used to identify putative replicated types for further assessment with our supervised framework (detailed above). New cell-type labels encompassing these replicate types (e.g., a combined Sst Chodl label containing Int1 (Zeisel), Sst Chodl (Tasic), and Sst Nos1 (Paul)) were generated for MetaNeighbor across random and GO sets, and for meta-analysis of differential expression. While only reciprocal top hits across laboratories were used to define putative replicate cell types, cross-validation within laboratories was performed to fill in AUROC scores for cell types within each laboratory.

### Differential expression

For each cell type within a dataset (defined by the authors’ original labeling), differential gene expression was calculated using a one-sided Wilcoxon rank-sum test, comparing gene expression within a given cell type to all other cells within the dataset (e.g., Zeisel_Int1 vs. all other Zeisel interneurons). Meta-analytic *P* values were calculated for each putative replicated type using Fisher’s method^59^, and then a multiple hypothesis test correction was performed with the Benjamini–Hochberg method^60^. Top differentially expressed genes were those with an adjusted meta-analytic FDR < 0.001 and with log2 fold change >2 in each dataset. All differential expression data for putative replicated subtypes can be found in Supplementary Data 3. Details regarding the generation of *Ptn*-CreER transgenic mice, immunostaining and imaging may be found in Paul et al. The image in panel 5c was taken at the same time as those presented in Supplementary Fig. 6 of that paper, but this image has never been published.

### Data availability

All scripts, data, and detailed directions to run MetaNeighbor in R can be found on our Github page^56^. Accession codes for publicly available data analyzed in this study can be found in Supplementary Table 1 and Supplementary Table 2.

## Electronic supplementary material

Supplementary Information

Description of Additional Supplementary Files

Supplementary Data 1

Supplementary Data 2

Supplementary Datat 3
