# Supplementary material for: Characterizing the replicability of cell types defined by single cell RNA-sequencing data using MetaNeighbor
Source: Nat Commun. 2018 Feb 28;9:884. doi: 10.1038/s41467-018-03282-0 (PMC5830442; doi:10.1038/s41467-018-03282-0)
Supplement: Supplementary file 1 — Supplementary Information [file 41467_2018_3282_MOESM1_ESM.pdf]

Supplementary Material

Crow *et al.*, "Characterizing the replicability of cell types defined by single cell RNA-sequencing data using MetaNeighbor"

Supplementary Figure 1 2

Supplementary Figure 2 3

Supplementary Table 1 5

Supplementary Table 2 7

Supplementary Table 3 8

Supplementary Note 1: Additional Use-cases 9

Human pancreas 9

Endocrine cells 9

Non-endocrine cells 9

Mouse whole retina vs. bipolar-enriched Drop-seq 10

Mouse bipolar-enriched Drop-seq vs. targeted bipolar deep scRNA-seq – data alignment 10

Mouse bipolar-enriched Drop-seq vs. targeted bipolar deep scRNA-seq – supervised analysis across gene sets from GO 11

Mouse pyramidal neuron subtypes 11

Supplementary Note 2 12

On highly variable gene set selection 12

Supplementary References 13

Supplementary Figure 1

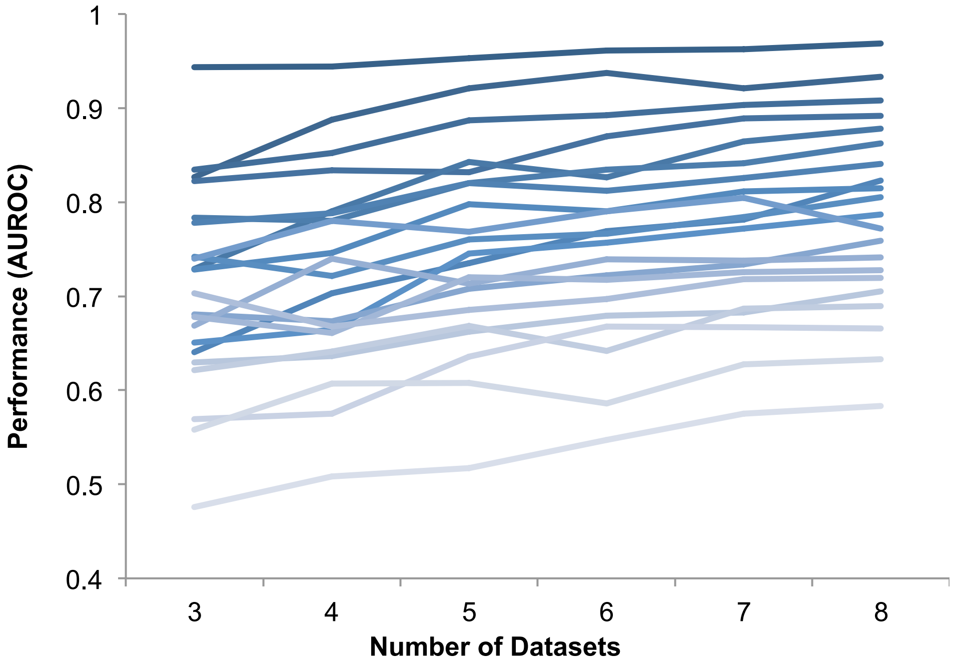

Supplementary Figure 1 – Data aggregation improves performance. The mean AUROC score for 21 representative gene sets is plotted against the number of datasets used to discriminate between neurons and non-neuronal cells (task one). For each gene set and each number of datasets, we randomly sampled through combinations of datasets 10 times, then plotted the average across these 10 permutations. While some gene sets are always more discriminative than others, AUROCs generally improve with additional training data.

Supplementary Figure 2

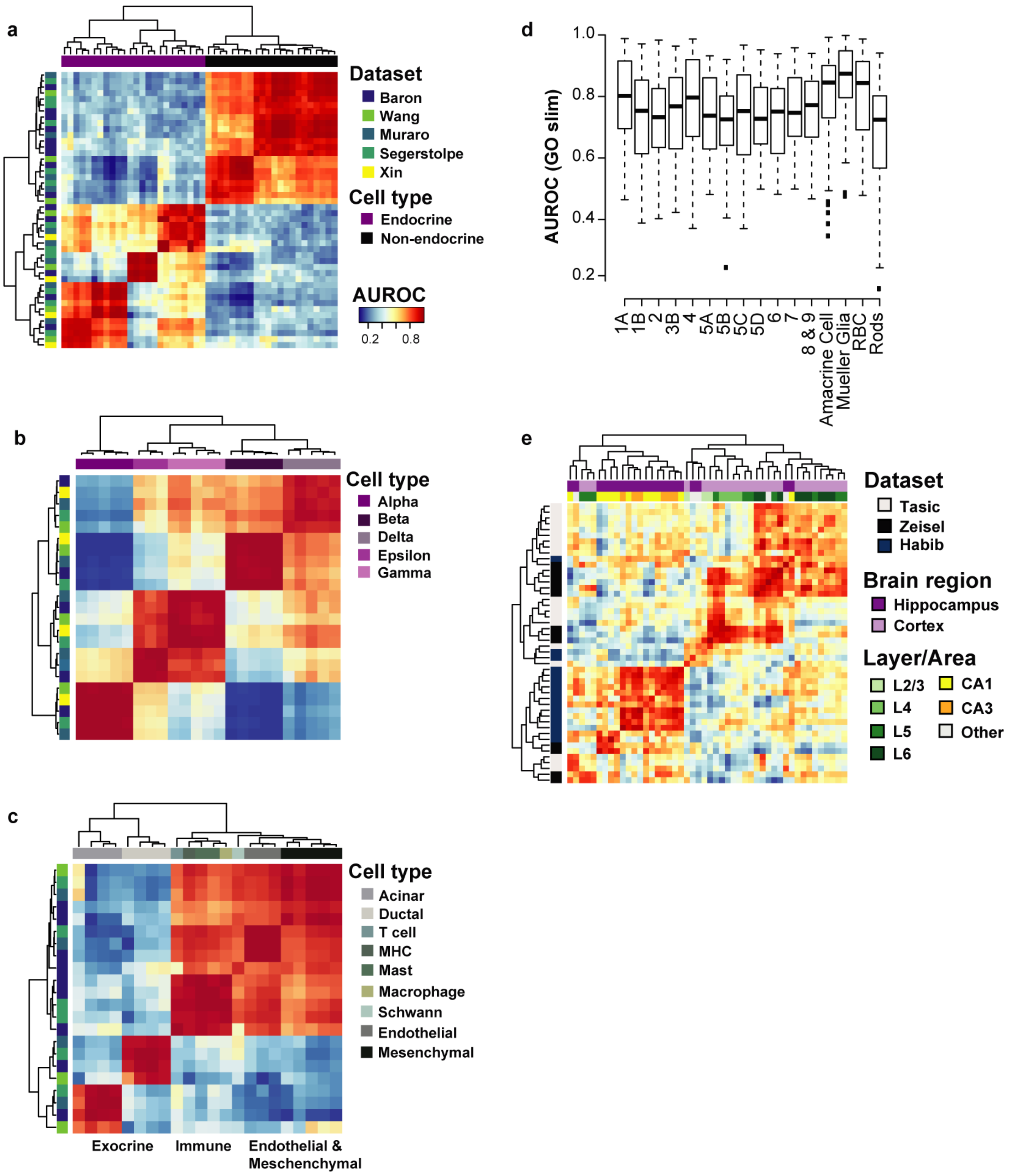

Supplementary Figure 2 – Additional use-cases, detailed in Supplementary Note 1

a - Heatmap of AUROC scores between pancreas cell types based on the highly variable gene set (HVG). Dendrograms were generated by hierarchical clustering of Euclidean distances using average linkage (used throughout). Row and column colors indicate data origin and broad cell class. Endocrine and non-endocrine cells are found in two distinct colors. No clustering based on data origin or technology is observed. **b -** Heatmap of AUROC scores between endocrine cell types of the pancreas based on HVGs. AUROCs and rows are labeled as in **a**. Column colors indicate endocrine cell types. Perfect replicability is observed, regardless of technological differences between datasets. **c -** Heatmap of AUROC scores between non-endocrine cell types of the pancreas based on HVGs. AUROCs and rows are labeled as in **a**. Column colors indicate cell types. Perfect replicability is observed between acinar, ductal, endothelial and mesenchymal cell types. Different immune cell classes were characterized across the four studies, indicating that comparison to a more comprehensive set of immune cell types would be informative. **d** – Box plots of GO slim performance for cell types from Shekhar *et al*. As for all cases within the main text, high AUROCs are observed across many GO groups. **e -** Heatmap of AUROC scores between pyramidal subtypes based on HVGs. Row colors indicate datasets and column colors show brain region, cortical layer or hippocampal area. AUROC color scale as in **a**. Clustering of AUROC score profiles shows a separation of cortical and hippocampal subtypes.

Supplementary Table 1

Related to Figures 2 and 3, sample information for MetaNeighbor validation tasks and empirical modeling.

GSE ID	First Author	Author Cell type ID	Label Task 1	Label Task 2	Samples
**GSE56638**	Dueck	Cortex; Pyramidal neuron	neuron	excitatory	19
**GSE56638**	Dueck	Dorsal Raphe; 5-HT neuron	neuron	NA	22
**GSE56638**	Dueck	Heart; Cardiomyocyte	non	NA	19
**GSE56638**	Dueck	Hippocampus; Pyramidal neuron	neuron	excitatory	18
**GSE56638**	Dueck	Interscapular brown adipose; Adipocyte	non	NA	13
**GSE59739**	Usoskin	NF	neuron	NA	139
**GSE59739**	Usoskin	NoN	non	NA	40
**GSE59739**	Usoskin	NP	neuron	NA	164
**GSE59739**	Usoskin	PEP	neuron	NA	65
**GSE59739**	Usoskin	TH	neuron	NA	230
**GSE60361**	Zeisel	astrocytes ependymal	non	NA	217
**GSE60361**	Zeisel	endothelial-mural	non	NA	223
**GSE60361**	Zeisel	interneurons	neuron	inhibitory	290
**GSE60361**	Zeisel	microglia	non	NA	90
**GSE60361**	Zeisel	oligodendrocytes	non	NA	817
**GSE60361**	Zeisel	pyramidal CA1	neuron	excitatory	939
**GSE60361**	Zeisel	pyramidal SS	neuron	excitatory	399
**GSE63472**	Macosko	Horizontal cells	neuron	NA	119
**GSE63472**	Macosko	RGC	neuron	NA	220
**GSE63472**	Macosko	Amacrine cells	neuron	NA	2562
**GSE63472**	Macosko	Rods	neuron	NA	1307
**GSE63472**	Macosko	Cones	neuron	NA	777
**GSE63472**	Macosko	Bipolar cells	neuron	NA	2469
**GSE63472**	Macosko	Muller glia	non	NA	477
**GSE63472**	Macosko	Astrocytes	non	NA	22
**GSE63472**	Macosko	Fibroblasts	non	NA	33
**GSE63472**	Macosko	Vascular endothelium	non	NA	117
**GSE63472**	Macosko	Pericytes	non	NA	17
**GSE63576**	Li	lumbar DRG neuron	neuron	NA	204
**GSE70844**	Fuzik	cortical neuron	neuron	NA	76
**GSE71585**	Tasic	Astrocyte	non	NA	43
**GSE71585**	Tasic	Endothelial Cell	non	NA	29

**GSE71585**	Tasic	GABA-ergic Neuron	neuron	inhibitory	761
**GSE71585**	Tasic	Glutamatergic Neuron	neuron	excitatory	812
**GSE71585**	Tasic	Microglia	non	NA	22
**GSE71585**	Tasic	Oligodendrocyte	non	NA	38
**GSE71585**	Tasic	Oligodendrocyte Precursor Cell	non	NA	22
**GSE71585**	Tasic	Unclassified	non	NA	13
**GSE75413**	Hanchate	Early Immature Neuron	neuron	NA	30
**GSE75413**	Hanchate	Late Immature Neuron	neuron	NA	16
**GSE75413**	Hanchate	Mature Neuron	neuron	NA	27
**GSE75413**	Hanchate	Progenitor	non	NA	11
**GSE85721**	Habib	CA1	NA	excitatory	119
**GSE85721**	Habib	CA2	NA	excitatory	1
**GSE85721**	Habib	CA3	NA	excitatory	49
**GSE85721**	Habib	DG	NA	excitatory	453
**GSE85721**	Habib	Ependymal	NA	NA	4
**GSE85721**	Habib	GABAergic	NA	inhibitory	111
**GSE85721**	Habib	Glia	NA	NA	74
**GSE85721**	Habib	Non	NA	NA	113

Total neuron	***11665***
Total non	***2263***
Total excitatory	***2809***
Total inhibitory	***1162***

Supplementary Table 2

Related to Figure 4, interneuron subtypes

GSE ID	First Author	Author Cell type ID	Samples	Mean detected genes
**GSE60361**	Zeisel	Int1	12	4389
**GSE60361**	Zeisel	Int10	21	3764
**GSE60361**	Zeisel	Int11	10	3943
**GSE60361**	Zeisel	Int12	21	4630
**GSE60361**	Zeisel	Int13	15	4110
**GSE60361**	Zeisel	Int14	22	4762
**GSE60361**	Zeisel	Int15	18	3290
**GSE60361**	Zeisel	Int16	20	4555
**GSE60361**	Zeisel	Int2	24	4834
**GSE60361**	Zeisel	Int3	10	4129
**GSE60361**	Zeisel	Int4	15	3918
**GSE60361**	Zeisel	Int5	20	4938
**GSE60361**	Zeisel	Int6	22	4572
**GSE60361**	Zeisel	Int7	23	4233
**GSE60361**	Zeisel	Int8	26	4922
**GSE60361**	Zeisel	Int9	11	3710
**GSE71585**	Tasic	Igtp	10	5072
**GSE71585**	Tasic	Ndnf_Car4	31	4865
**GSE71585**	Tasic	Ndnf_Cxcl14	35	4782
**GSE71585**	Tasic	Pvalb_Cpne5	14	4885
**GSE71585**	Tasic	Pvalb_Gpx3	63	5323
**GSE71585**	Tasic	Pvalb_Obox3	20	5721
**GSE71585**	Tasic	Pvalb_Rspo2	31	5868
**GSE71585**	Tasic	Pvalb_Tacr3	71	5933
**GSE71585**	Tasic	Pvalb_Tpbg	18	5124
**GSE71585**	Tasic	Pvalb_Wt1	58	5806
**GSE71585**	Tasic	Smad3	13	5493
**GSE71585**	Tasic	Sncg	9	5577
**GSE71585**	Tasic	Sst_Cbln4	68	5411
**GSE71585**	Tasic	Sst_Cdk6	19	5313
**GSE71585**	Tasic	Sst_Chodl	41	5986
**GSE71585**	Tasic	Sst_Myh8	41	4984

**GSE71585**	Tasic	Sst_Tacstd2	14	5736
**GSE71585**	Tasic	Sst_Th	19	5116
**GSE71585**	Tasic	Vip_Chat	48	4683
**GSE71585**	Tasic	Vip_Gpc3	50	4490
**GSE71585**	Tasic	Vip_Mybpc1	29	4414
**GSE71585**	Tasic	Vip_Parm1	45	4429
**GSE71585**	Tasic	Vip_Sncg	14	5076
**GSE92522**	Paul	ChC	80	8103
**GSE92522**	Paul	Pv	127	9561
**GSE92522**	Paul	Som-Nos1	136	9220
**GSE92522**	Paul	Sst-Mtnt	62	8437
**GSE92522**	Paul	Vip-Cck	64	9292
**GSE92522**	Paul	Vip-CR	63	7024

Supplementary Table 3

Estimated MetaNeighbor run times - Laptop (OSX 10.10.4, 1.6 GHz, 8GB RAM, R 3.3.2, Rstudio 0.99.446)

Experiments	Cell types	Samples	Gene sets	Time (s)
**2**	1	100	10	0.1
**2**	10	100	10	0.5
**2**	10	100	100	1.7
**2**	10	100	1000	17.5
**2**	1	1000	10	9
**10**	1	1000	10	9
**2**	10	1000	10	12
**2**	10	1000	100	93
**2**	10	1000	1000	979
**2**	10	10000	10	3653

The major determinant of run time is the number of cells to be assessed, followed by the number of gene sets. Speed-ups are possible with parallelization and installing libraries such as MRAN.

Supplementary Note 1: Additional Use-cases

All supporting data for the following use-cases may be found in Supplementary Figure 2 and Supplementary Data 2.

Human pancreas

To date, six single cell RNA-seq studies have been performed on the human pancreas[1-6]. Five of these used whole pancreas, while one enriched for islet cells only[1]. Across studies, a range of techniques were employed in the preparation of single cell RNA-seq libraries including inDrop[7], a droplet-based 3'-targeted in vitro transcription method; flow sorting cells into 384-well plates followed by full-length Smart-Seq2[8]; and the Fluidigm C1 system[9]. In spite of these technical differences, there are strong prior expectations of known cell markers and types in the pancreas, making it an ideal testing ground for MetaNeighbor. As a general note, it is useful to distinguish between the alignment of data where cell types are likely to overlap, and the validation of 'putative cell types', i.e., when cell types are speculative or rare. The former task seeks to find matches between each pair of datasets (high scores between each pair of datasets), while the latter asks if there is independent evidence for a given cell type in any dataset (good hit for a cell type in at least one other dataset). In this case, we will be aiming to find matches across all pairs of datasets.

In order to assess the replicability of the cell types across the five whole pancreas studies, we downloaded gene expression data and cell labels from the website of Dr. Martin Hemberg[10] in July of 2017. Gene names were aligned in R and only cells with valid cell type labels were retained for analysis (i.e., cells labeled "contaminated", "dropped", "unknown" etc., were removed). This yielded a matrix containing 15558 genes and 14805 cells. A breakdown of the number of cells per type and study can be found in Supplementary Data 2.

MetaNeighbor across all labeled cell types provided an interesting view onto more global patterns of transcriptional similarity, with non-endocrine cells showing strong internal consistency with respect to the endocrine cells of the pancreas, but more subtle differential signal among them (Supplementary Figure 2a). This suggests that a hierarchical approach, where we examine each of these cell classes in turn, may be useful, allowing for the selection of a more precise feature set and more refined cell type controls. This is similar to the approach in the main text, where we first examine differences between inhibitory and excitatory neurons, and then look within inhibitory neurons for replicability of types/subtypes.

Endocrine cells

In our analysis of the endocrine celltypes, we identified a set of 115 HVG using the get_variable_genes function from MetaNeighbor. Using this gene set in combination with pairwise testing and training, we found nearly perfect replicability (AUROCs of 1) for endocrine cell types in the five studies, despite major differences in technology and transcriptome coverage (Supplementary Figure 2b and Supplementary Data 2). Notably, and in accordance with the results from our empirical model, performance was equally high for rare epsilon cells (incidence 0.1-0.5%) as for more common delta cells (3-12%) and gamma cells (5-13%).

Non-endocrine cells

To analyze the non-endocrine cell types, we identified a set of 300 HVG to use for pairwise testing and training, only 8 of which were also found in the HVG set for the endocrine types (RGS2, LMCD1, NEDD9, GGCT, SOCS2, PNP, PRSS23, DNAJB9). Interestingly, when we looked at all cell types in the pancreas it was difficult to see the distinction between exocrine cell types and others (Figure 2c). Now acinar and ductal are found to be both replicable and distinct from one another, and from the non-exocrine related types. Among the

non-exocrine cells, we see high replicability of endothelial and mesenchymal cells. However, immune cells appear to have broad similarity to one another. From the labeling it appears that researchers have recognized varying immune populations (e.g. only the Baron data has a separate T cell type). We would suggest that these cell types would be usefully compared to more comprehensive immune cell datasets.

Mouse whole retina vs. bipolar-enriched Drop-seq

The cell architecture of the retina has been extensively characterized making it an attractive target for single cell RNA-seq. Two prominent papers describing the transcriptional diversity of retinal cell types have been published[11,12]. The first paper aimed to achieve a high-throughput sampling of the retina, whereas the second was a more targeted approach to sample bipolar neurons. The whole retina analysis resulted in an approximately accurate representation of the cell type mixture within the tissue, with ~80% of cells classified as rods or cones, while ~7% were bipolar cells that could be classified into 8 bipolar neuron subtypes. As a result of the targeted sampling approach the authors delineated 14 bipolar neuron clusters, 13 of which represented a unique subtype. To compare the results of these two studies, the authors performed two analyses: first, they re-clustered the bipolar cells from the whole retina using the same approach used for the bipolar cell-only analysis; second, they built a random forest classifier from the bipolar cell data, and compared the results of the new clustering analysis to the identities predicted by the algorithm. They found that 2/8 of the whole retina clusters were uniquely identified in the bipolar cell data (cone bipolar cells and rod bipolar cells), but that the remaining six clusters contained a mix of two or more subtypes.

After downloading data from GEO (GSE63472) and from the Broad Institute's Single Cell Data Portal[12] and removing cells expressing fewer than 1000 unique genes, we calculated AUROCs for all pairs of bipolar cells from the two experiments using MetaNeighbor and the highly variable gene set. Notably, we discovered the exact overlaps that were described in the original analyses (Supplementary Data 2), though in some cases this means that a single cluster from the Macosko analysis has a very high AUROCs for two or more clusters from the Shekhar analysis. This provides some guidance for the interpretation of pairwise scores. If scores are very high between one cell type and multiple others, then the selection of reciprocal best matches will only capture a fraction of the true replicability. MetaNeighbor scores are a jumping-off point for further investigation, providing clues about the structure of the data, without requiring re-clustering or the entrainment of machine learning classifiers.

Mouse bipolar-enriched Drop-seq vs. targeted bipolar deep scRNA-seq – data alignment

Within the Shekhar paper about bipolar cell heterogeneity, the authors also compare results between Drop-seq and two targeted deeper sequencing approaches, making use of transgenic mouse lines that express GFP from specific promoters, *Kcng4* and *Vsx2*. Cell labels in the *Kcng4* and *Vsx2* experiments were defined based on the Drop-seq profiles using the Random Forest classifier described above (NB labels for *Kcng4* and *Vsx2* cells were supplied by the authors upon request). Re-analysis of this classification using MetaNeighbor can be considered a positive control for the method, as we know that we should have perfect 1:1 cluster matches across pairs of datasets. A breakdown of the number of cells per type and dataset can be found in Supplementary Data 2.

In a head-to-head comparison between the Drop-seq data and the *Kcng4* data across the cell types represented in both, we find perfect replicability using the reciprocal top hit as our criterion (Supplementary Data 2). This includes the BC4 and BC5C types, each of which were represented with a single cell in the *Kcng4* data. Similarly, for the *Vsx2* data we find perfect replicability of all 15 types (2669 HVG), including the single rod photoreceptors (Supplementary Data 2). This demonstrates the flexibility and robustness of MetaNeighbor. Without performing any correction to account for differences in sequencing depth or batch

effects, we are able to quickly identify top overlaps between these technically divergent datasets, even when cells are extremely rare.

Mouse bipolar-enriched Drop-seq vs. targeted bipolar deep scRNA-seq – supervised analysis across gene sets from GO

Because there are known correspondences between the three bipolar cell datasets (Drop-seq, *Vsx2* and *Kcng4*), we can also use MetaNeighbor to see how cell type replicability scores change when different gene sets are used. Similar to the results from the interneuron, excitatory/inhibitory and neuron/non-neuronal comparisons, high mean AUROC scores are observed for every cell type (~0.8 across all gene sets, Supplementary Figure 2d). Notably, similar GO functions are high performing for these cells as for the tasks described in the main text (e.g., transmembrane transport). The top GO group for each cell type is listed in Supplementary Data 2.

Mouse pyramidal neuron subtypes

The heterogeneity of pyramidal neurons is undisputed, but the organizing principles are still debated, with some suggesting that identity is discrete and modular[13,14] and others purporting that identities are more likely to be described by expression gradients or spectra[15]. With MetaNeighbor we are able to quantitatively assess the degree to which pyramidal subtypes defined by scRNA-seq replicate across diverse datasets. If cell types are discrete and modular, we would expect to see sharp differences, with some types showing very strong similarity to one another, and strong dissimilarities to other types.

To compare pyramidal neuron scRNA-seq datasets we permuted through all combinations of subtypes as testing and training data using the highly variable gene set. As with the interneurons, this analysis confronted differences in clustering specificity between datasets, with the Zeisel dataset defining clusters at a high level (layers), and the Habib and Tasic datasets defining multiple sub-layer clusters, which necessarily limits the resolution of replicability that can be assessed. In total, we found that a small fraction of subtypes corresponded across datasets using reciprocal best match as our criterion for replication (10/48, ~21%) yielding five putative subtypes (Supplementary Data 2).

The AUROC score heatmap was generally less modular than the heatmap of interneuron scores (Supplementary Figure 2e). The most prominent feature was that types from the hippocampus and cortex tended to cluster separately from one another. Within each region-specific cluster, some layer- or area-specific clustering was observed but it was not completely consistent. Particular discrepancy was observed between the cortical layer 5 subtypes which showed more similar AUROC score profiles to the hippocampal subtypes than to other deep layer types (Tasic L5b_Cdh13, L5_Chrna6, L5b_Tph). These were also the same subtypes that Tasic *et al.* found no match for in their marker gene analysis A limited retrograde tracing analysis included within the Tasic paper indicated that projection neurons with ipsilateral thalamic projections could be identified as one of the two L5b types, and L5b types have previously been classified as having corticofugal projections[16]. CA1 neurons also project subcortically, and though we cannot confirm that this is the reason for their transcriptional similarity without further experimental validation, we believe this is an interesting and unusual prediction that would not be likely to be tested without the comprehensive testing scheme of MetaNeighbor.

Supplementary Note 2

On highly variable gene set selection

Within the main text and throughout Supplementary Note 1 we highlighted the utility of using highly variable genes to identify overlaps across datasets without requiring prior knowledge of marker genes. This is a common strategy for unsupervised feature selection within individual studies, and so to generalize across studies we require that genes are highly variable within each dataset, in essence prioritizing jointly present 'markers'. Of course, other strategies are possible, such as the use of the union of highly variable genes, or the use of highly variable genes from training data only.

To investigate the effects of these other feature selection strategies, we re-analyzed data from Tasic, Zeisel and Habib to distinguish between inhibitory and excitatory neurons (as for Figures 2B and 3). While performance is high across all gene sets, we find that performance for the union performs comparably to picking the worst performing gene set from an individual dataset ($AUROC_{union}$=0.89, $AUROC_{Habib}$=0.85), and that the intersect performs about as well as picking the best gene set from an individual dataset ($AUROC_{intersect}$=0.97, $AUROC_{Tasic}$=0.99). Using the HVG from a given dataset does not improve performance for that dataset itself when it is held-out for testing, but often improves predictions for other data.

Supplementary References

1 Li, J. *et al.* Single-cell transcriptomes reveal characteristic features of human pancreatic islet cell types. *EMBO reports* **17**, 178-187, doi:10.15252/embr.201540946 (2016).

2 Wang, Y. J. *et al.* Single-Cell Transcriptomics of the Human Endocrine Pancreas. *Diabetes* **65**, 3028-3038, doi:10.2337/db16-0405 (2016).

3 Baron, M. *et al.* A Single-Cell Transcriptomic Map of the Human and Mouse Pancreas Reveals Inter- and Intra-cell Population Structure. *Cell Systems* **3**, 346-360.e344, doi:10.1016/j.cels.2016.08.011 (2016).

4 Muraro, Mauro J. *et al.* A Single-Cell Transcriptome Atlas of the Human Pancreas. *Cell Systems* **3**, 385-394.e383, doi:10.1016/j.cels.2016.09.002 (2016).

5 Segerstolpe, A. *et al.* Single-Cell Transcriptome Profiling of Human Pancreatic Islets in Health and Type 2 Diabetes. *Cell metabolism* **24**, 593-607, doi:10.1016/j.cmet.2016.08.020 (2016).

6 Xin, Y. *et al.* RNA Sequencing of Single Human Islet Cells Reveals Type 2 Diabetes Genes. *Cell metabolism* **24**, 608-615, doi:10.1016/j.cmet.2016.08.018.

7 Klein, A. M. *et al.* Droplet barcoding for single-cell transcriptomics applied to embryonic stem cells. *Cell* **161**, 1187-1201, doi:10.1016/j.cell.2015.04.044 (2015).

8 Picelli, S. *et al.* Smart-seq2 for sensitive full-length transcriptome profiling in single cells. *Nature methods* **10**, 1096-1098, doi:10.1038/nmeth.2639 (2013).

9 Pollen, A. A. *et al.* Low-coverage single-cell mRNA sequencing reveals cellular heterogeneity and activated signaling pathways in developing cerebral cortex. *Nature biotechnology* **32**, 1053-1058, doi:10.1038/nbt.2967 (2014).

10 Hemberg, M. <https://hemberg-lab.github.io/scRNA.seq.datasets/human/pancreas/> (2017).

11 Macosko, E. Z. *et al.* Highly Parallel Genome-wide Expression Profiling of Individual Cells Using Nanoliter Droplets. *Cell* **161**, 1202-1214, doi:10.1016/j.cell.2015.05.002 (2015).

12 Shekhar, K. *et al.* Comprehensive Classification of Retinal Bipolar Neurons by Single-Cell Transcriptomics. *Cell* **166**, 1308-1323.e1330, doi:10.1016/j.cell.2016.07.054 (2016).

13 Zeisel, A. *et al.* Brain structure. Cell types in the mouse cortex and hippocampus revealed by single-cell RNA-seq. *Science (New York, N.Y.)* **347**, 1138-1142, doi:10.1126/science.aaa1934 (2015).

14 Habib, N. *et al.* Div-Seq: Single-nucleus RNA-Seq reveals dynamics of rare adult newborn neurons. *Science (New York, N.Y.)* **353**, 925-928, doi:10.1126/science.aad7038 (2016).

15 Cembrowski, M. S. *et al.* Spatial Gene-Expression Gradients Underlie Prominent Heterogeneity of CA1 Pyramidal Neurons. *Neuron* **89**, 351-368, doi:10.1016/j.neuron.2015.12.013 (2016).

16 Molyneaux, B. J., Arlotta, P., Menezes, J. R. L. & Macklis, J. D. Neuronal subtype specification in the cerebral cortex. *Nat Rev Neurosci* **8**, 427-437, doi:10.1038/nrn2151 (2007).
