# Supplementary material for: Characterizing the replicability of cell types defined by single cell RNA-sequencing data using MetaNeighbor
Source: Nat Commun. 2018 Feb 28;9:884. doi: 10.1038/s41467-018-03282-0 (PMC5830442; doi:10.1038/s41467-018-03282-0)
Supplement: Supplementary file 2 — Description of Additional Supplementary Files [file 41467_2018_3282_MOESM2_ESM.pdf]

Description of Additional Supplementary Files

File Name: Supplementary Data 1
Description: **Interneuron AUROC scores**. **Worksheet 1** - AUROC scores between all pairs of interneuron subtypes from the Paul, Tasic and Zeisel datasets as shown in Figure 4. **Worksheet 2** - AUROC scores between the Tasic and Zeisel datasets only, with legends to indicate previously reported overlaps.

File Name: Supplementary Data 2
Description: **Data associated with the additional use-cases from Supplementary Note 1. Worksheets 1-3 –** Human pancreas cell type replicability analysis. Sheet 1 - The number of cells of each type across the five datasets considered. Sheet 2 - Reciprocal top hits and AUROC scores >0.95 across the endocrine cells only. Cell types are labeled with a short name followed by the first initial of the last author's name (e.g., alpha_b indicates alpha cells from the Baron dataset). Sheet 3 - All reciprocal top hits and AUROC scores >0.95 across the non-endocrine cells. Cells are labeled as in Sheets 1 and 2. **Worksheets 4-8** – Bipolar cell type replicability analysis. Sheet 4 – AUROC scores between Macosko and Shekhar bipolar cell types. Sheet 5 - The number of the cells per type across the three datasets from Shekhar *et al*. Sheet 6 - All reciprocal top hits between the Drop-seq and *Kcng4* Smart-seq data. Sheet 7– All reciprocal top hits between the Drop-seq and *Vsx2* Smart-seq data. Sheet 8 – Top GO functions for each cell type after performing MetaNeighbor across all three datasets. **Worksheet 9** - Reciprocal top hits from the pyramidal neuron replicability analysis.

File Name: Supplementary Data 3
Description: **Interneuron differential expression meta-analysis.** Tables report meta-analytic p-values with multiple hypothesis test correction (BH_adj_Fisher_p) for each putatively replicated subtype, as well as fold changes for each subtype within its own experiment. Genes that have previously been reported as markers and those with high fold changes in all datasets are indicated.
